# Arbuscular mycorrhizal fungi and rhizobium facilitate nitrogen uptake and transfer in soybean/maize intercropping system

**DOI:** 10.3389/fpls.2015.00339

**Published:** 2015-05-13

**Authors:** Lingbo Meng, Aiyuan Zhang, Fei Wang, Xiaoguang Han, Dejiang Wang, Shumin Li

**Affiliations:** ^1^Resource and Environmental College, Northeast Agricultural UniversityHarbin, China; ^2^Heilongjiang Provincial Key University Laboratory of Cold Area Vegetable Biology, Northeast Agricultural UniversityHarbin, China; ^3^Department of Life Science, Harbin UniversityHarbin, China

**Keywords:** arbuscular mycorrhizal fungi, nitrogen uptake, nitrogen transfer, ^15^N, rhizobium, soybean/maize intercropping

## Abstract

The tripartite symbiosis between legumes, rhizobia and mycorrhizal fungi are generally considered to be beneficial for the nitrogen (N) uptake of legumes, but the facilitation of symbiosis in legume/non-legume intercropping systems is not clear. Therefore, the aims of the research are as follows: (1) to verify if the dual inoculation can facilitate the N uptake and N transfer in maize/soybean intercropping systems and (2) to calculate how much N will be transferred from soybean to maize. A pot experiment with different root separations [solid barrier, mesh (30 μm) barrier and no barrier] was conducted, and the ^15^N isotopic tracing method was used to calculate how much N transferred from soybean to maize inoculated with arbuscular mycorrhizal fungi (AMF) and rhizobium in a soybean (*Glycine max* L.cv. Dongnong No. 42)/maize (*Zea mays* L.cv. Dongnong No. 48) intercropping system. Compared with the *Glomus mosseae* inoculation (*G.m.*), *Rhizobium* SH212 inoculation (SH212), no inoculation (NI), the dual inoculation (SH212+*G.m.*) increased the N uptake of soybean by 28.69, 39.58, and 93.07% in a solid barrier system. N uptake of maize inoculated with both *G. mosseae* and rhizobium was 1.20, 1.28, and 1.68 times more than that of *G.m.*, SH212 and NI, respectively, in solid barrier treatments. In addition, the amount of N transferred from soybean to maize in a dual inoculation system with a mesh barrier was 7.25, 7.01, and 11.45 mg more than that of *G.m.*, SH212 and NI and similarly, 6.40, 7.58, and 12.46 mg increased in no barrier treatments. Inoculating with both AMF and rhizobium in the soybean/maize intercropping system improved the N fixation efficiency of soybean and promoted N transfer from soybean to maize, resulting in the improvement of yield advantages of legume/non-legume intercropping.

## Introduction

Legume and non-legume intercropping cultivation has been widely encouraged in sustainable agriculture because it has the potential to improve the yield significantly and allow plants to use soil N more efficiently (Eaglesham et al., 1981; Li et al., 2001, 2011; Hauggaard-Nielsen et al., 2009; Gao et al., 2014), which is beneficial for reducing the amount of chemical fertilizer supplies and has positive consequences on the environment (Lekberg and Koide, 2005; Pelzer et al., 2012). N could be used efficiently in the intercropping system because the N fixed by legumes can be transferred to companion species, and this part of N is a crucial source for the non-nodulated crop’s growth and development (Moyer-Henry et al., 2006). For example, Fujiu et al. (1990) have found that the amount of N transferred to sorghum (*Sorghum bicolor Moench* cv. Yuldjirushi) accounted for 32–58% of its N uptake in a soybean (*Glycine max* L. cv. Kurosengoku)/sorghum intercropping system. A substantial amount of N is transferred in different communities including N_2_-fixed and non-N_2_ fixed plants (Chu et al., 2004; Sierra and Daudin, 2010; Isaac et al., 2012; Frankow-Lindberg and Dahlin, 2013; Jamont et al., 2013; Chapagain and Riseman, 2014). In addition, inoculating rhizobium can significantly increase the yield and N uptake of wheat (*Triticum aestivum* L. cv. Long 17) and faba bean (*Vicia faba* L. cv. Linxia Dacandou) and further improve the intercropping advantages. This has been confirmed by Xiao et al. (2006), who inoculated rhizobia strain NM353 for faba bean in faba bean/wheat intercropping system. Fang et al. (2009) showed that the biomass and grain yield of faba bean (*V. faba* L. cv. Lincan No. 2) and maize (*Zea mays* L. cv. Zhongdan No. 2) and the number of faba bean nodules were increased similarly when inoculated with rhizobia strain GS374 in the faba bean/maize intercropping system. Several studies also indicated that inoculating both AMF and rhizobium can promote the growth of crops and improve the yield and nutrient uptake of crops (Lekberg and Koide, 2005; Antunes et al., 2006; Varennesa and Goss, 2007; Tajini et al., 2011; Abd-Alla et al., 2014). AMF is considered to be of great importance in plant symbiosis and promoting nutrient uptake, especially P (Li et al., 2004; Pasqualini et al., 2007; Xiao et al., 2010; Tajini et al., 2011; Abd-Alla et al., 2014). The mycelium can extend to the area outside the rhizosphere, connect roots with the surrounding soil microhabitats and enlarge the area that roots have to absorb nutrients (He et al., 2003). Thus, water and nutrients can be transported by the huge hyphae network to be finally absorbed by plants (Tobar et al., 1994; Vassilev et al., 2001; Yao et al., 2001; He et al., 2003). The N transfer in intercropping systems is assumed to be enhanced if N fixation by legumes can be improved by inoculation with AMF and rhizobium, which have the potential to enhance plant productivity. However, the effects of inoculating both rhizobium and AMF in legume/non-legume intercropping systems on N transfer are currently uncertain. Therefore, the objectives of our study are as follows: (1) to verify if the dual inoculation can facilitate N uptake and N transfer in a maize/soybean intercropping system, (2) to use the ^15^N isotopic tracing method to calculate how much N will be transferred between maize and soybean intercropping under the inoculation of both rhizobium and AMF.

## Materials and Methods

### Experiment Design

**FIGURE 1:**
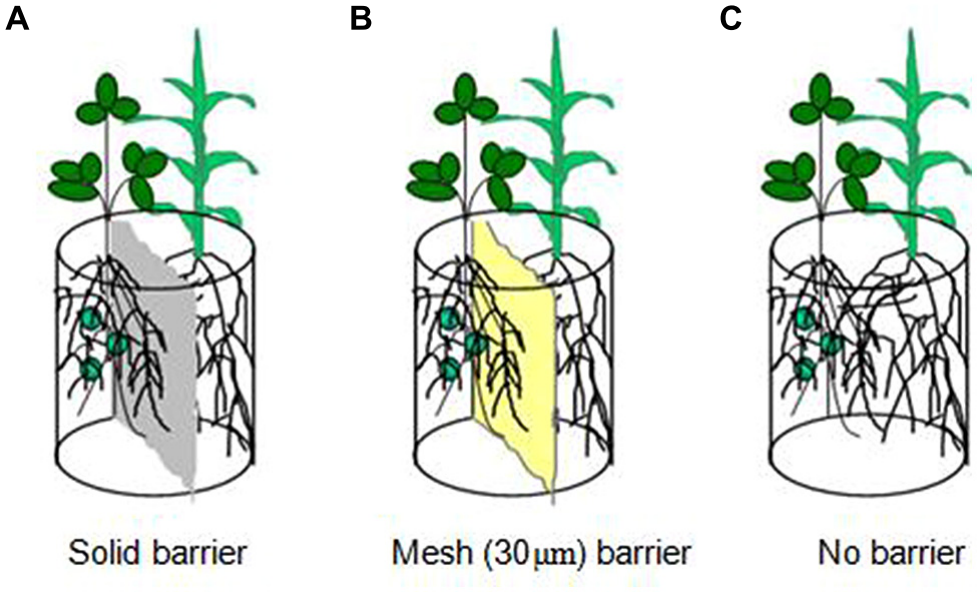
**Schematic diagram of the root separation in pots. (A)** is solid barrier, **(B)** is mesh (30 ţm) barrier and **(C)** is no barrier.

A pot experiment was conducted at a greenhouse in Northeast Agricultural University in China. Three root separation patterns between soybean and maize were designed (**Figure 1**) to study N uptake facilitation in an intercropping system. They were as follows: (1) solid barrier, roots were separated by hard plastic sheet (0.5 mm) and had no root contact or material exchange; (2) mesh barrier, roots were separated by a 30-μm nylon mesh and had no contact but water, nutrient and hyphae were allowed to exchange and permeate; (3) no barrier, which allowed for complete contact between the roots of soybean and maize. Plastic pots (3 kg capacity) were cut in the middle, separated into two compartments and then reconstructed for solid barrier and mesh barrier patterns.

### Plant Growth Medium

The soil used in the experiment contained 6.28 g kg^-1^ of organic matter, 1.2 g kg^-1^ of total N, 30.4 mg kg^-1^ of available N, 5.9 mg kg^-1^ of Olsen P and 167 mg kg^-1^ of available K. The soil was sieved (2 mm) and sterilized at 120°C for 2 h to eliminate the AMF spores, and 1.4 kg of the soil was then put into each compartment of the plastic pot. Then, basal nutrients were added in solution to the pot (mg kg^-1^ soil): N 100 mg (NH_4_NO_3_), P 80 mg (KH_2_PO_4_), K 150 mg (K_2_SO_4_), Mg 50 mg (MgSO_4_⋅7H_2_O), the microelement Fe (FeSO_4_⋅7H_2_O), Mn (MnCl_2_), Cu (CuCl_2_), Zn (ZnSO_4_⋅7H_2_O), and Mo [(NH_4_)_4_MoO_4_] 5 mg and were then thoroughly mixed, and each compartment was provided with 200 ml of water.

### Seeding and Inoculating

Seeds of soybean (*Glycine max* L. cv. Dongnong No. 42) and maize (*Z. mays* L. cv. Dongnong No. 48) were sterilized by immersion in 10% H_2_O_2_ for 30 min before seeding. Then, four seeds of soybean were sown into one compartment of the pot on May 19th, and two seeds of maize were sown into the other compartment on May 24th for intercropping. When the seeds germinated, the soybean seedlings were thinned to two plants per compartment and the maize seedlings were thinned to one plant for further growth.

The experiment involved four microbial treatments: inoculating with *Bradyrhizobium japonicum* SH212 (SH212), inoculating with *Glomus mosseae* (*G.m.*), dual inoculation (both of *Bradyrhizobium japonicum* SH212 and *G. mosseae*, SH212+*G.m.*) and NI as a control. The total was 12 treatments (3 barriers × 4 inoculations) with four replicates for each treatment. The rhizobium used was *B. japonicum* SH212 obtained from the rhizobium research group of Northeast Agricultural University in China, and the AMF used was *G. mosseae*, originating from a mycorrhizal research group of China Agricultural University. At sowing, 30 g per compartment of AMF inoculum and 15 ml per compartment of rhizobium (density was 8.2 × 10^8^/ml) were thoroughly mixed with the soil for inoculated treatments. Because the AMF inoculum consisted of the AMF spores, sand and colonized root fragments, the non-AMF inoculated treatments were amended with steam-sterilized inoculum. All of the pots were placed randomly.

### ^15^N Labeling

When the soybean was undergoing pod growth, an isotopic labeling experiment was conducted utilizing (^15^NH_4_)_2_SO_4_, enriched with 99% ^15^N. Before labeling, a PVC board was inserted between soybean and maize shoots, and a plastic film with two layers of filter paper on top was set on the surface of soil to prevent pollution from isotopic N. A microinjector (25 μL) was used to inject 10 μL of 88 mM (^15^NH_4_)_2_SO_4_ solution into the petioles of soybeans every day. Each labeling was replicated four times. Soybean petioles were labeled for 9 days. The plants without labeling were used as a control to examine the natural ^15^N abundance.

### Sampling and Analysis

Plants were harvested on July 18th. The shoots were first cut off at ground level and separated by their different inoculated treatments and root separation patterns; then, the whole soil in the pot was removed and placed on a sieve with 1-mm mesh to pick up the nodules. The roots of soybean and maize were then washed with running tap water and separated the same way as shoots. All of the fresh nodules (including the nodules removed from the soil) of the soybean roots were counted and recorded.

Samples of fresh roots were cut into segments of ∼1 cm and mixed thoroughly. One gram of fresh root was randomly collected to estimate the root-colonization of AMF. The root samples were stained with Trypan blue and faded with lactic acid and glycerin; then, 30 pieces of root segments were observed under a visible light microscope to estimate AMF colonization (Phillips and Hayman, 1970). Every root segment was defined according to the standard of the mycorrhizal infection. Next, “MYCOCALC” software was used to calculate arbuscular mycorrhizal colonization (Trouvelot et al., 1986).

The shoots and the remaining fresh roots were dried at 70°C to a constant weight after killing the enzymatic activity at 105°C for 0.5 h. The plant samples were digested with H_2_SO_4_-H_2_O_2_ methods for N analysis, and the total N content of plants was measured using the Kjeldahl procedure.

The ^15^N abundance of shoots was determined using a DELTA PLUS XP isotope ratio mass spectrometer (FINNIGAN).

### Calculating and Statistical Analysis

N transfer was calculated as follows:

(1) $$N\%=N_{1}\%-N_{c}\%$$

Where *N*% indicates the atomic percentage of ^15^N excess of the plant (maize or soybean), *N*_l_% indicates the atomic percentage of ^15^N in the labeled plant and *N*_c_% indicates the atomic percentage of ^15^N in the control plant;

(2) $$N_{t}\%=\frac{N_{m}\times N_{m}\%}{N_{m}\times N_{m}\%+N_{s}\times N_{s}\%}\times 100$$

Where *N*_t_% indicates the percentage of N uptake by soybean transfer to associated maize, *N*_m_ and *N*_s_ indicate the uptake of maize and soybean (mg/pot) and *N*_m_% and *N*_s_% indicate the atomic percentage of ^15^N excess in maize and soybean, respectively;

(3) $$N_{t}=N_{t}\%\times N_{s}$$

Where *N*_t_ indicates the amount of N that soybean transferred to maize (mg/pot); and

(4) $$N_{o}\%=\frac{N_{t}}{N_{m}}\times 100$$

Where *N*_o_% indicates the percentage of transferred N that occupies the maize N uptake.

Statistical analysis was performed using SPSS19.0 software (SPSS, Inc., Chicago, IL, USA). The differences of treatments were compared using the least significant difference (LSD) and the *t*-test at a significance level of *p* ≤ 0.05 after analysis of variance (ANOVA).

## Results

### Biomass

Dual inoculation treatment (SH212+*G.m.*) significantly increased soybean total biomass by 67.70% with a solid barrier, by 70.40% with a mesh barrier and by 72.80% with no barrier patterns compared with NI treatment (**Table 1**). Rhizobium SH212 and *G. mosseae* as single inocula also significantly facilitated soybean’s growth; the biomass of the soybean shoots and roots were significantly higher than that of the NI group in all three root separation patterns (**Table 1**). However, no significant difference was observed in soybean shoots, roots, and total biomass between SH212 and *G.m*. treatments (**Table 1**). The root separation had no significant influence on soybean biomass in each inoculated and non-inoculated treatment (**Table 1**). Additionally, no interaction was found between root separation and inoculation treatments.

**Table 1:** The biomass of shoots and roots of soybean and maize inoculated with AMF and rhizobium with solid barrier, mesh barrier and no barrier (g/pot).

Treatments		Soybean			Maize		
		Shoot	Root	Total	Shoot	Root	Total
Solid barrier	NI	4.36 ± 0.04 c^a^A^b^	1.12 ± 0.04 cA	5.48 ± 0.07 cA	8.48 ± 0.03 cC	5.02 ± 0.05 cC	13.5 ± 0.08 cC
	SH212	5.33 ± 0.02 bA	2.18 ± 0.03 bA	7.50 ± 0.05 bA	9.50 ± 0.02 bB	6.03 ± 0.05 bB	15.53 ± 0.04 bB
	*G.m.*	5.36 ± 0.04 bA	2.22 ± 0.07 bA	7.58 ± 0.11 bA	9.51 ± 0.03 bB	6.05 ± 0.03 bB	15.56 ± 0.07 BC
	SH212+ *G.m.*	6.67 ± 0.13 aA	2.53 ± 0.01 aA	9.19 ± 0.04 aA	10.79 ± 0.04 aB	7.31 ± 0.05 aB	18.10 ± 0.08 aB
Mesh barrier	NI	4.30 ± 0.06 cA	1.14 ± 0.08 cA	5.44 ± 0.13 cA	8.60 ± 0.02 cB	5.15 ± 0.03 cB	13.75 ± 0.03 cB
	SH212	5.37 ± 0.02 bA	2.24 ± 0.02 bA	7.61 ± 0.03 bA	9.64 ± 0.02 bAB	6.24 ± 0.03 bA	15.88 ± 0.06 bA
	*G.m*.	5.44 ± 0.03 bA	2.25 ± 0.01 bA	7.69 ± 0.08 bA	9.65 ± 0.03 bB	6.22 ± 0.02 bA	15.87 ± 0.05 bB
	SH212+ *G.m.*	6.72 ± 0.03 aA	2.55 ± 0.04 aA	9.27 ± 0.07 aA	10.95 ± 0.03 aA	7.53 ± 0.03 aA	18.48 ± 0.06 aAB
No barrier	NI	4.21 ± 0.03 cA	1.12 ± 0.04 cA	5.33 ± 0.06 cA	8.70 ± 0.05 cA	5.28 ± 0.02 cA	13.98 ± 0.07 cA
	SH212	5.36 ± 0.03 bA	2.21 ± 0.04 bA	7.57 ± 0.06 bA	9.83 ± 0.05 bA	6.30 ± 0.02 bA	16.12 ± 0.03 bA
	*G.m.*	5.34 ± 0.06 bA	2.19 ± 0.07 bA	7.53 ± 0.13 bA	9.90 ± 0.06 bA	6.31 ± 0.02 bA	16.21 ± 0.07 bA
	SH212+ *G.m.*	6.70 ± 0.08 aA	2.51 ± 0.04 aA	9.21 ± 0.08 aA	11.09 ± 0.06 aA	7.66 ± 0.06 aA	18.75 ± 0.06 aA
Inoculation		^∗^	^∗^	^∗^	^∗∗^	^∗∗^	^∗∗^
Root separation		ns	ns	ns	^∗∗^	^∗∗^	^∗∗^
Inoculation × root separation		ns	ns	ns	ns	ns	ns

The data above are expressed as the means ± SD (*n* = 4).

^a^Mean values of inoculated treatments with same root barrier followed by different lower case letters (a, b, c, and d) are significantly different (*p* < 0.05).

^b^Mean values of three root barriers with the same inoculation treatment followed by different capital letters (A, B, and C) are significantly different (*p* < 0.05).

ns, indicates no significant difference; ^∗^ and ^∗∗^ mean significant at 5 and 1% levels, respectively.

With regard to maize, the highest biomass of maize was also observed in the group treated with SH212+*G.m.* and was significantly higher than that of SH212, *G.m.* and NI treatments in every root separation pattern (**Table 1**). Rhizobium SH212 inoculation increased maize shoot and root biomass by 12.99 and 19.32%, respectively, compared with NI in the no root separation pattern (**Table 1**). In addition, *G. mosseae* inoculation also increased both shoot biomass and root biomass (**Table 1**). Moreover, a trend was observed that maize biomass in the no barrier pattern was significantly higher than that of mesh barrier or solid barrier patterns whether inoculated or not (**Table 1**). For example, the total biomass of maize in the no barrier system increased by 3.56 and 1.67% compared with solid barrier and mesh barrier systems under non-inoculated treatments (**Table 1**). However, no interaction was found between inoculation and root separation treatments.

### The AMF Colonization Rate

**FIGURE 2:**
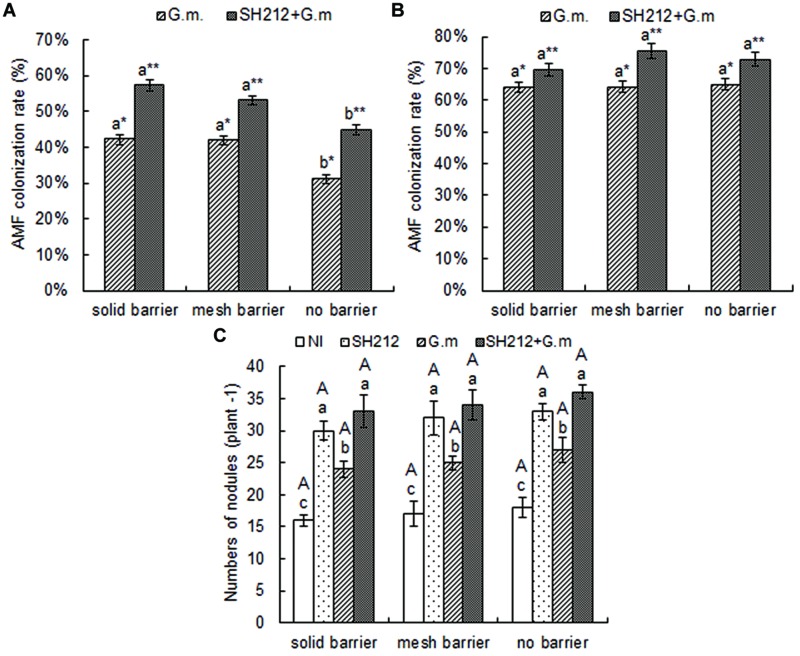
**Arbuscular mycorrhizal fungi colonization rates of soybean **(A)** and maize **(B)** inoculated with AMF and rhizobium with three root separation patterns.** Bars with different lower case letters indicate significant differences between different root barriers in the same inoculated treatments (*p* < 0.05). Asterisks (^∗^ and ^∗∗^) indicate significant differences between different inoculated treatments in the same root separation patterns (*p* < 0.05). **(C)** is the number of soybean nodules with three root separation patterns and inoculating AMF and rhizobium. The NI, SH212, *G.m.,* and SH212+*G.m.* in the figures represent NI treatment, SH212 inoculation treatment, *Glomus mosseae* inoculation treatment and both SH212 and *G. mosseae* inoculation treatment, respectively. Bars with different lower case letters indicate significant differences between different inoculated treatments in the same root separation patterns, and bars with different capital letters indicate significant differences between different root separation patterns in the same inoculated treatment (*p* < 0.05). Means ± SD of four replicates.

No AMF colonization was found in the roots of soybean and maize not inoculated with *G. mosseae*, and the AMF colonization rate was 0 (data not shown). The AMF colonization rate of soybean was increased when plants were inoculated with *G. mosseae*, and the increase was more significant when rhizobium SH212 was also inoculated concurrently (**Figure 2A**). Hence, AMF colonization increased by 35.55, 26.73, and 43.59% in solid barrier, mesh barrier and no barrier systems when co-inoculated with SH212 (**Figure 2A**). The AMF colonization rate of soybean plants was significantly increased through intercropping with maize. In addition, the AMF colonization rate of maize in a no barrier system was higher than that of a solid barrier both in *G.m.* and SH212+*G.m.*, but no significant difference was observed (**Figure 2B**).

### The Number of Soybean Nodules

The number of soybean root nodules increased as a result of inoculation of microsymbionts (**Figure 2C**). The *G. mosseae* inoculation alone (*G.m.*) and rhizobium SH212 inoculation alone (SH212) significantly increased the number of nodules. However, the greatest increase was observed when both *G. mosseae* and rhizobium SH212 were inoculated (**Figure 2C**). Compared to NI, the number of nodules was increased 2.6, 2.0, and 2.0 times with dual inoculation in solid barrier, mesh barrier and no barrier system, respectively (**Figure 2C**). A small but insignificant increase in the amount of soybean root nodules was found in the no barrier pattern compared with the mesh barrier and solid barrier systems regardless of inoculation status (**Figure 2C**).

### N Concentration and N Uptake

SH212+*G.m.* treatment significantly increased the N concentrations in soybean shoots and roots by 13.72 and 18.47%, respectively, compared with NI treatment in no barrier patterns (**Table 2**). In addition, a uniform facilitation was found in maize shoots and roots, as the N concentrations increased by 28.34 and 34.94%, respectively (**Table 2**). The root separation patterns had little influence on the N concentration of soybean shoots and roots (**Table 2**). However, separating maize from soybean plants by a plastic sheet significantly decreased the N concentration of maize shoots (by 5.04–13.25%) compared with no barrier patterns in all inoculated treatments (**Table 2**).

**Table 2:** Shoots and roots N concentration (mg/g) and N uptake (mg/pot) of soybean and maize inoculated with AMF and rhizobium with three roots separation patterns.

	Treatments	Soybean			Maize		
		Shoot		Root		Shoot		Root	
		N concentration	N uptake	N concentration	N uptake	N concentration	N uptake	N concentration	N uptake
Solid barrier	NI	19.93 ± 0.50 b^a^A^b^	86.88 ± 1.46 cA	18.87 ± 0.80 cA	21.06 ± 1.36 cA	11.78 ± 0.50 cB	99.87 ± 4.20 dB	14.15 ± 0.20 cA	70.99 ± 1.44 cB
	SH212	19.96 ± 0.20 bA	106.32 ± 1.15 bcA	19.74 ± 0.60 bcA	42.96 ± 1.15 bA	13.77 ± 0.30 bB	130.88 ± 2.69 cB	15.39 ± 0.40 bA	92.71 ± 1.92 bB
	*G.m.*	21.19 ± 0.40 abA	113.57 ± 1.84 bA	21.71 ± 0.80 abA	48.33 ± 2.91 bA	15.27 ± 0.10 aB	145.21 ± 1.76 bB	15.46 ± 0.20 bA	93.52 ± 1.93 bB
	SH212+*G.m.*	22.63 ± 0.80 aA	150.83 ± 5.37 aA	22.78 ± 0.90 aA	57.59 ± 2.43 aA	14.73 ± 0.50 abB	158.89 ± 5.62 aB	17.35 ± 0.10 aB	126.86 ± 1.79 aB
Mesh barrier	NI	19.71 ± 0.40 bA	84.78 ± 1.76 cA	18.90 ± 0.20 cA	21.49 ± 5.30 cA	11.99 ± 0.40 bB	103.02 ± 2.99 cB	14.22 ± 0.10 cA	73.23 ± 0.91 cAB
	SH212	20.77 ± 1.00 abA	111.53 ± 5.35 bA	20.90 ± 0.40 bA	46.78 ± 1.15 bA	14.27 ± 0.50 aAB	137.54 ± 5.41 bB	15.55 ± 0.20 bA	96.98 ± 0.95 bAB
	*G.m.*	20.29 ± 1.20 abA	110.34 ± 6.48 bA	21.57 ± 0.50 bA	49.48 ± 1.41 bA	15.86 ± 0.50 aAB	153.00 ± 5.68 abA	15.71 ± 0.20 bA	97.68 ± 1.34 bAB
	SH212+*G.m.*	22.52 ± 0.30 aA	151.30 ± 2.84 aA	22.80 ± 0.20 aA	58.21 ± 1.15 aA	15.33 ± 1.00 aAB	167.87 ± 10.70 aAB	18.85 ± 0.30 aAB	141.93 ± 2.54 aA
No barrier	NI	19.61 ± 0.30 bA	82.48 ± 1.70 cA	18.79 ± 0.30 cA	21.10 ± 0.85 cA	13.23 ± 0.20 dA	115.08 ± 1.65 dA	14.48 ± 0.50 bA	76.36 ± 2.26 cA
	SH212	20.22 ± 0.50 bA	108.27 ± 2.89 bA	19.85 ± 0.10 bcA	43.86 ± 0.63 bA	15.24 ± 0.10 cA	149.67 ± 0.68 cA	15.89 ± 0.80 bA	100.06 ± 4.91 bA
	*G.m.*	19.94 ± 0.70 bA	106.50 ± 4.08 bA	20.70 ± 0.70 bcA	45.24 ± 0.53 bA	16.08 ± 0.10 bA	159.14 ± 1.87 bA	15.90 ± 0.60 bA	100.31 ± 3.79 bA
	SH212+*G.m.*	22.30 ± 0.50 aA	149.45 ± 5.23 aA	22.26 ± 0.30 aA	55.97 ± 1.59 aA	16.98 ± 0.20 aA	188.31 ± 2.26 aA	19.54 ± 0.80 aA	149.61 ± 6.11 aA
Inoculation		^∗^	^∗^	^∗^	^∗^	^∗^	^∗∗^	^∗^	^∗∗^
Root separation		ns	ns	ns	ns	ns	^∗∗^	ns	^∗∗^
Inoculation × Root separation		ns	ns	ns	ns	ns	ns	ns	ns

The data above are expressed as the means ± SD (*n* = 4).

^a^Mean values of inoculated treatments with the same root barrier followed by different lower case letters (a, b, c, and d) are significantly different (*p* < 0.05).

^b^Mean values of three root barriers with the same inoculation treatment followed by different capital letters (A, B, and C) are significantly different (*p* < 0.05).

ns, indicates no significant difference; * and ** mean significant at 5 and 1% levels, respectively.

**FIGURE 3:**
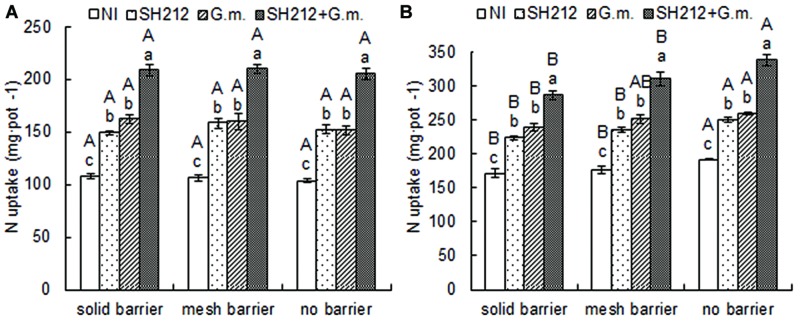
**Nitrogen uptake of the whole plant of soybean **(A)** and maize **(B)** inoculated with AMF and rhizobium and with three root separation patterns in a soybean/maize intercropping system.** The NI, SH212, *G.m.*, and SH212+*G.m.* in the figures represent NI treatment, SH212 inoculation treatment, *G. mosseae* inoculation treatment and both SH212 and *G. mosseae* inoculation treatment, respectively. Bars with different lower case letters indicate significant differences between different inoculated treatments in the same root separation pattern, and bars with different capital letters indicate significant differences between different root separation patterns in the same inoculated treatment (*p* < 0.05). Means ± SD of four replicates.

The highest N uptake of soybean was found with SH212+*G.m.* treatment in all three root separation patterns (**Table 2** and **Figure 3A**). No significant difference was observed in the N uptake of soybean shoots and roots between different root separation patterns (**Table 2**). For maize, both the root separation and microbial inoculation had significant effects on N uptake (**Table 2** and **Figure 3B**). Dual inoculation increased maize shoot N uptake by 59.93, 63.00, and 63.62% and root N uptake by 78.57, 93.87, and 96.08% compared with NI treatment in solid barrier, mesh barrier and no barrier patterns, respectively (**Table 2**). In addition, the N uptake of maize was significantly enhanced by intercropping with soybean, and the N uptake of the no barrier pattern was 12.01% higher than the solid barrier pattern under non-inoculated conditions (**Figure 3B**).

### The N Transfer in Soybean/Maize Intercropping Systems

The results of ^15^N labeling showed that *G. mosseae* and rhizobium SH212 inoculation alone enhanced the N transfer from soybean to maize in a soybean/maize intercropping system (**Table 3**). However, the more significant enhancement was observed in dual inoculation in mesh barrier and no barrier systems (**Table 3**). The amount of N transferred from soybean to maize (*N*_t_) of SH212+*G.m.* was 11.45 and 12.46 mg more than that of NI, and it was also significantly more than SH212 or *G.m.* alone in mesh barrier and no barrier patterns (**Table 3**). In addition, the transferred N from soybean to intercropped maize accounted for 3.13–6.01% of the N uptake of maize (**Table 3**). However, no significant difference was observed in the percentage of transferred N that occupied maize N uptake (*N*_o_%) between *G.m.* and SH212 (**Table 3**). The N transfer was also increased by intercropping. For example, the amount of N transferred from soybean to maize (*N*_t_) in a no barrier system was 19.63–43.33% more than that in a mesh barrier system (**Table 3**).

**Table 3:** Nitrogen transferred from the ^15^N labeled soybean to the associated maize with three root separation patterns and inoculation with AMF and rhizobium.

Treatments	*N*_t_%		*N*_t_ (mg/pot)		*N*_o_%	
	Mesh barrier	No barrier	Mesh barrier	No barrier	Mesh barrier	No barrier
NI	5.19 c^a^B^b^	7.57 cA	5.52 cB	7.84 bA	3.13 bB	4.10 bA
SH212	6.29 bB	8.36 bA	9.96 bB	12.72 aA	4.25 bB	5.09 aA
*G.m.*	6.08 bB	9.16 bA	9.72 bB	13.90 aA	3.88 bB	5.36 aA
SH212+ *G.m.*	8.10 aB	9.88 aA	16.97 aB	20.30 aA	5.48 aB	6.01 aA

The data above are expressed as the means (*n* = 4).

^a^Mean values of inoculated treatments with the same root barrier followed by different lower case letters are significantly different (*p* < 0.05).

^b^Mean values of three root barriers with the same inoculation treatments followed by different capital letters (A and B) are significantly different (*p* < 0.05).

## Discussion

The growth of maize plants in a no barrier system was facilitated greatly over those with a mesh barrier or solid barrier, regardless of the status of inoculation, confirming the yield advantage in maize/soybean intercropping systems in agreement with previous reports (Hauggaard-Nielsen and Jensen, 2005; Chapagain and Riseman, 2014). The biomass of soybean and maize inoculated with both AMF and rhizobium were more than that of NI in all root separation patterns, which illustrates that inoculating rhizobium and AMF can enhance the biological yield advantages of soybean and maize. This is consistent with our former research results that the biomass of soybean supplied with different phosphorus sources was improved significantly when inoculated AMF and rhizobium (Tong et al., 2009). Xiao et al. (2010) have found that inoculating AMF in upland rice (*Oryza sativa* ssp. Japonica Nipponbare) and mungbean (*Vigna radiata* L. cv. Chuanyuan) intercropping systems increased the biomass of mungbean by 288.8%. In addition, Mei et al. (2012) found that the average grain yields of faba bean (*V. faba* L.) and maize (*Z. mays* L.) increased by 30–197% and 0–31%, respectively, after inoculating with rhizobium in maize and faba bean intercropping systems in reclaimed desert soil. In our study, we inoculated both rhizobium and AMF in a soybean/maize intercropping system. The soybean and maize biomass was 21.66 and 16.32% higher than that of SH212 alone and 22.31 and 15.67% higher than that of *G.m.* alone in a no barrier pattern (**Table 1**). That suggested synergistic facilitation for yield advantage was observed in maize/soybean intercropping because of inoculating both AMF and rhizobium.

### Why Did Inoculating Rhizobium and AMF in Soybean/Maize Intercropping System Improve Growth of Maize and Soybean?

In our experiment, both AMF and rhizobium colonization independently increased the total biomass of soybean in solid barrier patterns compared with their respective controls, and the total biomass of soybean with dual inoculation was 1.68 times as much as that of NI (**Table 1**). We found synergistic effects of AMF and rhizobium on soybean growth, which was consistent with the results of Abd-Alla et al. (2014), who found that dual inoculation with rhizobium and AMF was more efficient for promoting growth of faba beans (*V. faba* L.). Rhizobium symbiosis is involved in the fixation of atmospheric N, whereas AMF improves the ability of a plant to absorb P and other nutrients (Li et al., 2006; Erman et al., 2011; Tajini et al., 2012; Pellegrino and Bedini, 2014). Our previous study found that maize overyielding in maize/faba bean or soybean intercropping resulted from its uptake of phosphorus mobilized by the acidification of the rhizosphere via fababean root by using mesh (permeable) and solid (impermeable) root barriers. The level of soybean to acidify rhizosphere is lower than faba bean (Li et al., 2007). The present study showed that N uptake by soybean inoculated with both AMF and rhizobium with no barriers was 1.98 times as much as that of the NI group (**Figure 3A**). Therefore, the increase in dry matter accumulation could be attributed to the incremental increase on nodulation, N fixation and nutrient acquisition.

In this experiment, we found that the N uptake of maize with no barriers was 8.63 and 12.01% more than that with mesh barriers or solid barriers under non-inoculated conditions, and 9.08 and 17.94% more under dual inoculated conditions (**Figure 3B**). In addition, the results showed that N transfer from soybean inoculated with both AMF and rhizobium to maize in no barrier and mesh barrier patterns increased 12.46 and 11.45 mg/pot compared with the NI group (**Table 3**), which means that the N transfer was improved due to the dual inoculation. Therefore, the biomass of maize was improved due to the increase of N uptake after intercropping with soybean and inoculating with AMF and rhizobium. This is in agreement with the results of Zarea et al. (2011), Larimer et al. (2014), and Pellegrino and Bedini (2014).

### AM Fungal Hyphae Contribute to N Transfer in Soybean/Maize Intercropping Systems

Arbuscular mycorrhizal fungi are important components in intercropping agrosystems (Li et al., 2009; Yan et al., 2014). In our study, N was transferred under non-inoculation conditions in mesh barrier patterns, but the rate and amount of N transferred in SH212+*G.m.* inoculations were 1.56 and 3.07 times more than that of the NI group (**Table 3**), which resulted from the improved AMF colonization rate of soybean and maize by inoculating with both rhizobium and AMF. The 30-μm nylon-net prevented the direct contact of the roots of soybean and maize but allowed hyphae to penetrate and link, and the hyphae enhanced the degree of contact of soybean and maize and the degree of contact of roots affected N transfer significantly, in agreement with Chu et al. (2004).

Many researchers suggested that there were two pathways for N transfer. One is a direct transfer that N fixed by legumes is transferred to associated non-N_2_ fixed plants via a mycorrhizal fungal hyphae network (Cardoso and Kuyper, 2006; Sierra and Nygren, 2006). The N concentration of legumes is generally higher than graminaceous; therefore, N could transfer to intercropped graminaceous along the gradient of concentration via hyphae (Chu et al., 2004). The other pathway is an indirect transfer, in which the residual and root exudates (Jalonen et al., 2009) of legumes release N to the rhizosphere when they decompose, and the mineralized inorganic N can then be absorbed by the intercropped graminaceous or mycorrhizal hyphae (Tomm et al., 1994; Johansen and Jensen, 1996; He et al., 2003). In our experiment, the rate and the amount of N transferred from soybean to maize were improved by microbial inoculations. Hence, no matter which way the N is transferred, the hyphae play an important role in N transfer from soybean to associated maize.

In addition, we found that inoculating rhizobium also promoted the growth of maize. Some studies have confirmed that PGPR were beneficial for plant growth, yield and crop quality (Zafar et al., 2012; Stefan et al., 2013; Güneş et al., 2014; Yadav and Verma, 2014). PGPRs could enhance asymbiotic N_2_ fixation and nutrient uptake and compete against detrimental microorganisms (Dey et al., 2004; Lucy et al., 2004; Khan, 2005; Yadav and Verma, 2014), which would be the reason that the growth of maize increased with rhizobium inoculation in our experiment.

Arbuscular mycorrhizal fungi and rhizobium establish beneficial symbiosis with legumes and enhance the advantage of intercropping, and the nutrient uptake and biomass of intercropped crops were significantly increased. Therefore, co-inoculation with both AMF and rhizobium should be considered for the sustainable development of the legume/graminaceous intercropping pattern.

## Conflict of Interest Statement

The authors declare that the research was conducted in the absence of any commercial or financial relationships that could be construed as a potential conflict of interest.

## Abbreviations

AMF: Arbuscular mycorrhizal fungi
N: nitrogen
NI: no inoculation
SH212: inoculating *Bradyrhizobium japonicum* SH212
*G.m.*: inoculating *Glomus mosseae*
SH212+*G.m.*: inoculating both *Bradyrhizobium japonicum* SH212 and *Glomus mosseae*
PGPR: plant growth-promoting rhizobacteria.
